# Supplementary material for: A timeline of symptom onset and disease progression in CLN3 disease
Source: Orphanet J Rare Dis. 2026 Jan 7;21:38. doi: 10.1186/s13023-025-04174-5 (PMC12871039; doi:10.1186/s13023-025-04174-5)
Supplement: Supplementary file 1 — Supplementary Material 1 [file 13023_2025_4174_MOESM1_ESM.docx]

**Supplementary Table 1. Estimated Data from Transformation of Reported Datasets.**

| Symptoms | |  | Ostergaard 2011^a^ | | Lebrun 2011^b^ | | Nielsen & Ostergaard 2013^b^ | | | | Arnsten 2019^b^ | | Abdennadher 2021^c^ | |
| --- | --- | --- | --- | --- | --- | --- | --- | --- | --- | --- | --- | --- | --- | --- |
|  |  |  | - | | - | | Female | | Male | | - | | - | |
|  |  |  | Reported | Estimated | Reported | Estimated | Reported | Estimated | Reported | Estimated | Reported | Estimated | Reported | Estimated |
| Cognitive decline | Mean |  |  |  | 7 | 7.24 |  |  |  |  |  |  |  |  |
|  | SD |  |  |  |  | 2.05 |  |  |  |  |  |  |  |  |
|  | Range |  |  |  | 4-12 |  |  |  |  |  |  |  |  |  |
|  | N |  |  |  | 25 |  |  |  |  |  |  |  |  |  |
| Epileptic seizures | Mean |  |  |  |  |  | 9.9 |  | 11.2 |  | 10.6 |  | 10 |  |
|  | SD |  |  |  |  |  | 2.6 |  | 2.9 |  |  |  |  | 5.04 |
|  | IQR |  |  |  |  |  |  |  |  |  |  |  | 6.8 |  |
|  | N |  |  |  |  |  | 16 |  | 19 |  | 22 |  | 14 |  |
| Complete blindness | Mean |  |  |  | 12.5 | 13.34 | 12.4 |  | 12.4 |  |  |  |  |  |
|  | SD |  |  |  |  | 3.61 | 1.9 |  | 2.1 |  |  |  |  |  |
|  | Range |  |  |  | 8.5-24.5 |  |  |  |  |  |  |  |  |  |
|  | N |  |  |  | 25 |  | 16 |  | 19 |  |  |  |  |  |
| Motor  decline | Mean |  |  |  | 10 | 10.11 |  |  |  |  |  |  |  |  |
|  | SD |  |  |  |  | 3.01 |  |  |  |  |  |  |  |  |
|  | Range |  |  |  | 5-16 |  |  |  |  |  |  |  |  |  |
|  | N |  |  |  | 25 |  |  |  |  |  |  |  |  |  |
| Speech and language impairment | Mean |  |  |  | 11 | 11.37 |  |  |  |  |  |  |  |  |
|  | SD |  |  |  |  | 4.08 |  |  |  |  |  |  |  |  |
|  | Range |  |  |  | 5-20 |  |  |  |  |  |  |  |  |  |
|  | N |  |  |  | 25 |  |  |  |  |  |  |  |  |  |
| Cardiac manifestations | Median |  | 18 |  |  |  |  |  |  |  |  |  |  |  |
|  | Mean |  |  | 17.86 | 17.7 |  |  |  |  |  |  |  |  |  |
|  | Range |  | 14-21 |  |  |  |  |  |  |  |  |  |  |  |
|  | SD |  |  | 1.77 | 7.3 |  |  |  |  |  |  |  |  |  |
|  | N |  | 29 |  | 16 |  |  |  |  |  |  |  |  |  |
| Loss of independent walking | Mean |  |  |  | 20.5 | 20.03 | 17 | 17 | 20.2 |  | 21.8 | 22.2 |  |  |
|  | Range |  |  |  | 11-27 |  | 13.8-20.6 |  | 14.3-22.1 |  | 18-29 |  |  |  |
|  | SD |  |  |  |  | 4.51 |  | 1.87 |  | 2.26 |  | 3.22 |  |  |
|  | N |  |  |  | 25 |  | 16 |  | 19 |  | 10 |  |  |  |
|  |  |  |  |  |  |  |  |  |  |  |  |  |  |  |
| Feeding difficulties | Mean |  |  |  |  |  | 19.8 | 19.8 | 24 | 23.81 |  |  |  |  |
|  | Range |  |  |  |  |  | 17.8-22.3 |  | 19.8-27.1 |  |  |  |  |  |
|  | SD |  |  |  |  |  |  | 1.23 |  | 1.99 |  |  |  |  |
|  | N |  |  |  |  |  | 16 |  | 19 |  |  |  |  |  |
|  |  |  |  |  |  |  |  |  |  |  |  |  |  |  |
| Death | Mean |  |  |  |  |  |  |  |  |  | 27.7 | 27.92 |  |  |
|  | SD |  |  |  |  |  |  |  |  |  |  | 5.47 |  |  |
|  | Range |  |  |  |  |  | from 20.9 |  | from 16.7 |  | 20-39 |  |  |  |
|  | N |  |  |  |  |  |  |  |  |  | 12 |  |  |  |

^a^ Mean and SD were estimated using the formula described by Cai et al.

^b^ We assumed a normal distribution and therefore that the reported means were an approximation of the median. The reported mean (approximated median) was then used with the reported range and N to estimate mean and SD using the formula described by Cai et al.

^c^ SD was estimated by dividing the IQR by 1.35

**SD**, standard deviation; **IQR**, interquartile range; **M**, male; **F**, female

**Supplementary Table 2: Combined Dataset for Core Symptoms of CLN3 Disease**

|  | **Studies with Mean (+/- SD)**  **Studies with transformed Mean (+/- SD)** | | | | **Sex Differences** | | | | | | |
| --- | --- | --- | --- | --- | --- | --- | --- | --- | --- | --- | --- |
|  | *Relates to Figure 1A-B* | | | | *Relates to Figure 1C* | | | | | | |
| Symptom | Weighted Mean | Weighted SD | Combined N | Number of Studies | Weighted Mean | | Weighted SD | | Combined  N | | Number of Studies |
|  |  |  |  |  | F | M | F | M | F | M |  |
| Vision  Loss | 6.1 | 1.6 | 254 | 4 | 6.4 | 5.6 | 1.3 | 1.3 | 54 | 63 | 2 |
| Behavioural changes | 8.5 | 3.9 | 194 | 2 |  |  |  |  |  |  |  |
| Cognitive decline | 9.3 | 3.1 | 219 | 3 |  |  |  |  |  |  |  |
| Epileptic seizures | 10.2 | 3.0 | 243 | 4 | 9.6 | 10.2 | 2.5 | 2.7 | 55 | 63 | 2 |
| Sleep disturbance | 11 | 6.1 | 111 | 1 |  |  |  |  |  |  |  |
| Complete blindness | 11.4 | 3.6 | 171 | 3 |  |  |  |  |  |  |  |
| Motor  decline | 11.0 | 3.8 | 108 | 2 |  |  |  |  |  |  |  |
| Speech and language impairment | 12.7 | 4.8 | 136 | 2 |  |  |  |  |  |  |  |
| Parkinsonian gait | 14.1 | 2.5 | 111 | 1 |  |  |  |  |  |  |  |
| Cardiac manifestations | 17.8 | 4.4 | 45 | 2 |  |  |  |  |  |  |  |
| Loss of independent walking | 19.5 | 3.2 | 70 | 3 |  |  |  |  |  |  |  |
| Feeding difficulties | 22.0 | 1.6 | 35 | 1 |  |  |  |  |  |  |  |
| Death | 22.4 | 4.4 | 95 | 2 |  |  |  |  |  |  |  |

**SD**, standard deviation; **M**, male; **F,** female
